# Supplementary material for: T-type voltage-gated channels, Na+/Ca2+-exchanger, and calpain-2 promote photoreceptor cell death in inherited retinal degeneration
Source: Cell Commun Signal. 2024 Feb 1;22:92. doi: 10.1186/s12964-023-01391-y (PMC10836022; doi:10.1186/s12964-023-01391-y)
Supplement: Supplementary file 2 — Additional file 1: Figure S1. Photoreceptor scRNA-Seq and effects of interventions targeting photoreceptor Ca2+-permeable channels. A) Differentially expressed genes (DEGs) in rd1 rod photoreceptors at post-natal day (P)13. B) DEGs in rd1 cone photoreceptors at P13. C) Effect of 10 µM BAPTA-AM and 40 µM SN-6 treatments on rd1*Cngb1-/- retinal cultures. TUNEL and the outer nuclear layer (ONL) thickness in treated retinas compared to Untr. rd1 specimens. D) Different interventions targeting Ca2+-permeable channels in wt retinal explant cultures. Scatter plots show percentage of TUNEL positive cells in ONL. Scale bars in C = 50 µM. Statistical significance was assessed using one-way ANOVA and Tukey’s multiple comparison post hoc test. Untr.: n=8 retinal explants from different animals; BAPTA: 5; L-cis: 4; CM4620: 6; SN-6: 7; D-cis: 6; TTA-A2: 3; DS5565: 4. Figure S2. Dose-response curves for BAPTA-AM, CM 4620, SN-6, TTA-A2, DS5565, and NA-184. A) Dose-response curve for BAPTA-AM in rd1 explant cultures. In the outer nuclear layer (ONL), 10, 25, and 50 µM BAPTA-AM significantly reduced calpain activity, PARP activity, and cell death as detected via the TUNEL assay. B) Different concentrations of CM4620 were tested in rd1 explant cultures. In the ONL, at concentrations of 20 µM and 60 µM, CM4620 significantly increased ONL calpain activity, PARP activity, and cell death, as assessed by the TUNEL assay. C) Dose-response for SN-6 in rd1 explant cultures. 20 µM and 40 µM SN-6 significantly reduced calpain activity, PARP activity, and cell death (TUNEL) in the ONL. B) Different concentrations of TTA-A2 were tested in rd1 explant cultures. In ONL, 10 µM TTA-A2 significantly reduced calpain activity, PARP activity, and cell death as assessed with the TUNEL assay. C) Dose-response for DS5565 in rd1 explant cultures. 15 µM DS5565 significantly reduced calpain activity, PARP activity, but not cell death (TUNEL) in the ONL. D) Dose-response for NA-184 in rd1 explant cultures. At c [file 12964_2023_1391_MOESM1_ESM.docx]

**SUPPLEMENTAL MATERIALS**


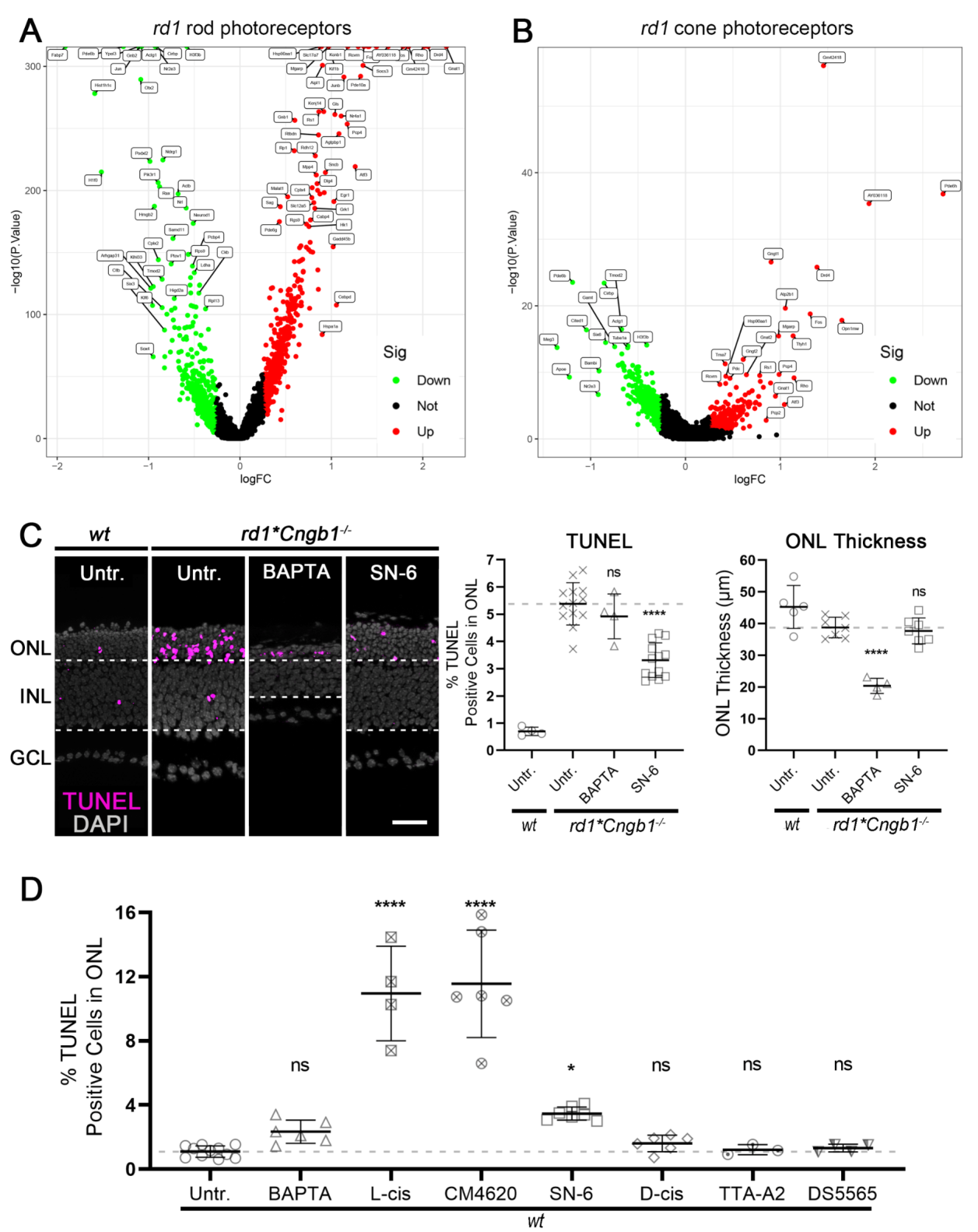


**Figure S1. Photoreceptor scRNA-Seq and effects of interventions targeting photoreceptor Ca^2+^-permeable channels. A**) Differentially expressed genes (DEGs) in *rd1* rod photoreceptors at post-natal day (P)13. **B**) DEGs in *rd1* cone photoreceptors at P13. **C**) Effect of 10 µM BAPTA-AM and 40 µM SN-6 treatments on *rd1*Cngb1^-/-^* retinal cultures. TUNEL and the outer nuclear layer (ONL) thickness in treated retinas compared to Untr. *rd1* specimens. **D**) Different interventions targeting Ca^2+^-permeable channels in *wt* retinal explant cultures. Scatter plots show percentage of TUNEL positive cells in ONL. Scale bars in C = 50 µM. Statistical significance was assessed using one-way ANOVA and Tukey’s multiple comparison *post hoc* test. Untr.: n=8 retinal explants from different animals; BAPTA: 5; L-cis: 4; CM4620: 6; SN-6: 7; D-cis: 6; TTA-A2: 3; DS5565: 4.


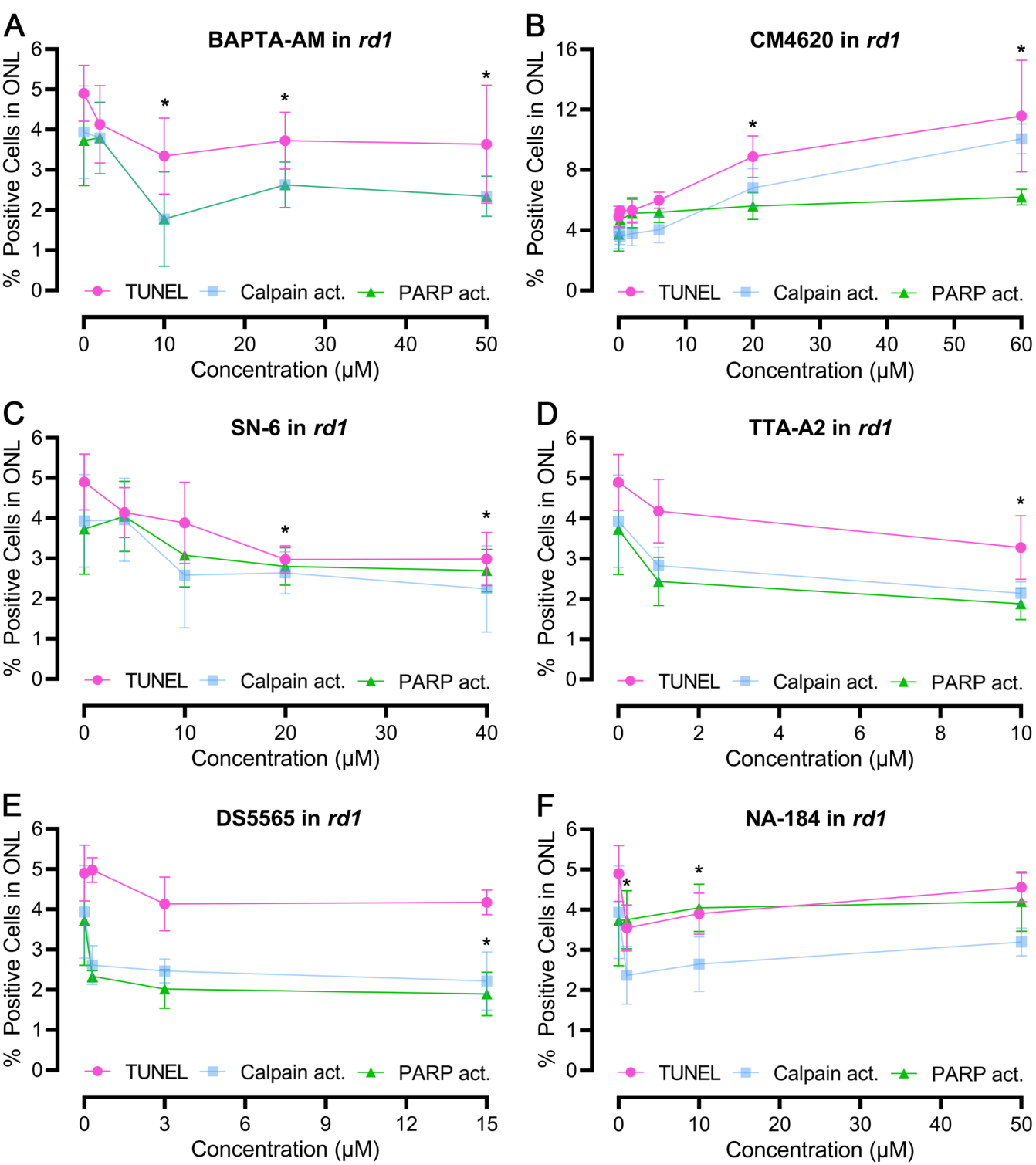


**Figure S2. Dose-response curves for BAPTA-AM, CM 4620, SN-6, TTA-A2, DS5565, and NA-184. A**) Dose-response curve for BAPTA-AM in *rd1* explant cultures. In the outer nuclear layer (ONL), 10, 25, and 50 µM BAPTA-AM significantly reduced calpain activity, PARP activity, and cell death as detected *via* the TUNEL assay. **B**) Different concentrations of CM4620 were tested in *rd1* explant cultures. In the ONL, at concentrations of 20 µM and 60 µM, CM4620 significantly increased ONL calpain activity, PARP activity, and cell death, as assessed by the TUNEL assay. **C**) Dose-response for SN-6 in *rd1* explant cultures. 20 µM and 40 µM SN-6 significantly reduced calpain activity, PARP activity, and cell death (TUNEL) in the ONL. **B**) Different concentrations of TTA-A2 were tested in *rd1* explant cultures. In ONL, 10 µM TTA-A2 significantly reduced calpain activity, PARP activity, and cell death as assessed with the TUNEL assay. **C**) Dose-response for DS5565 in *rd1* explant cultures. 15 µM DS5565 significantly reduced calpain activity, PARP activity, but not cell death (TUNEL) in the ONL. **D**) Dose-response for NA-184 in *rd1* explant cultures. At concentrations of 1 µM and 10 µM NA-184 significantly reduced ONL calpain activity and cell death (TUNEL) but did not decrease PARP activity. Statistical significance was assessed using one-way ANOVA and Tukey’s multiple comparison post hoc test.


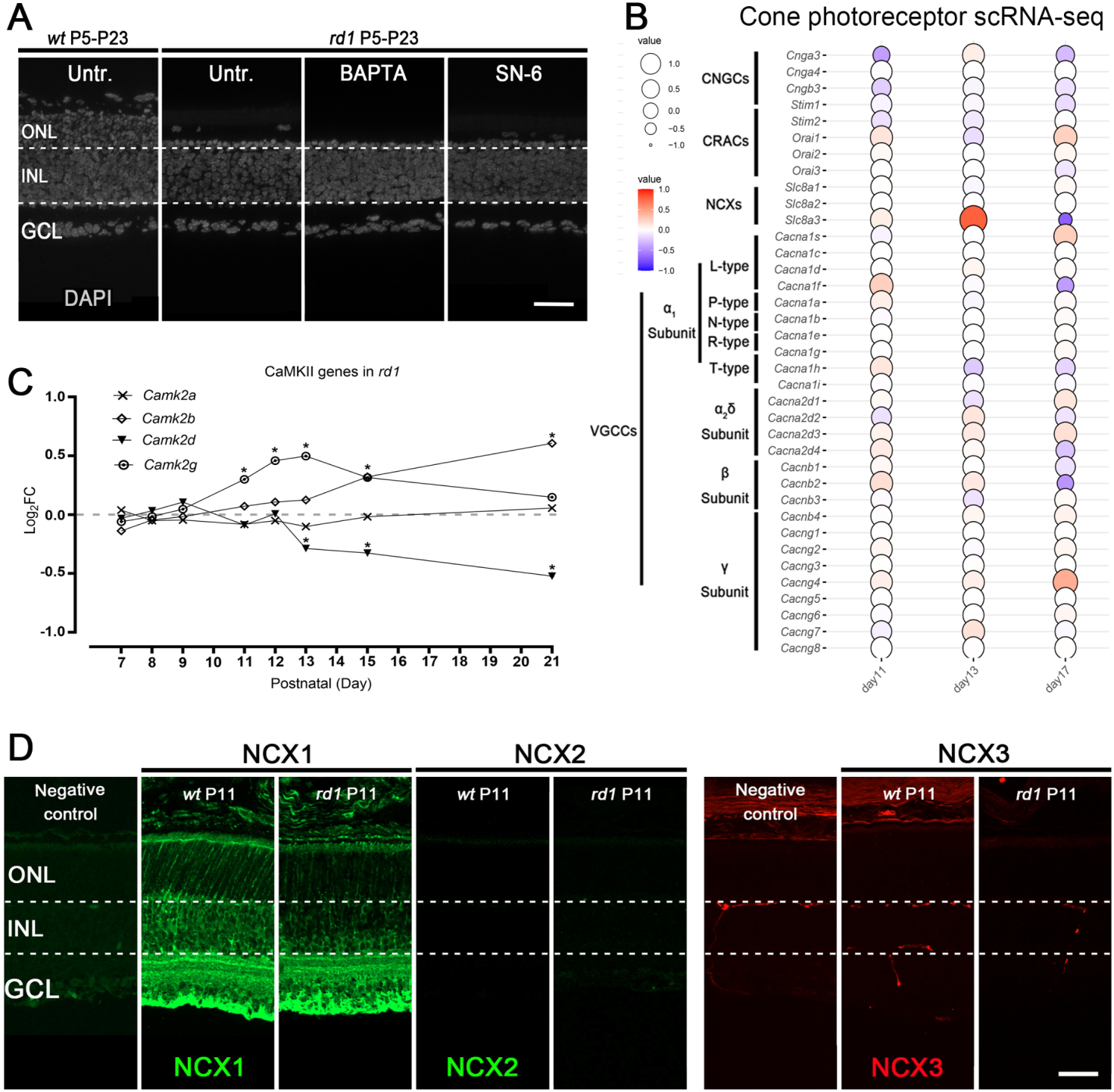


**Figure S3. Effects of interventions targeting photoreceptor Ca^2+^-permeable channels, bioinformatic analysis, and retinal NCX expression****. A**) Long-term treatment from P5 to P23, with 10 µM BAPTA and 40 µM SN-6, in *rd1* explant cultures, compared to untreated (Untr.) wild-type (*wt*) and *rd1* specimens. **B**) Balloon plot showing time-dependent expression changes (post-natal day (P) 11 to P17) of cyclic nucleotide-gated channel (CNGC), Ca^2+^-release activated channel (CRAC), Na^+^/Ca^2+^ exchanger (NCX), and voltage-gated Ca^2+^ channel (VGCC) in *rd1* cone photoreceptors. **C**) Analysis of Ca^2+^/calmodulin-dependent protein kinase II (CaMK2) gene expression during *rd1* photoreceptor degeneration. **D**) Immunostaining of Na^+^/Ca^2+^ exchanger (NCX) family. NCX1 was expressed in both inner and outer retina, while NCX2 and NCX3 were not detected. Occasional staining for NCX3 in retinal blood vessels relates to the use of anti-mouse secondary antibodies and (false positive) detection of IgG.

**Supplemental Table 1 – Differentially expressed genes (DEGs) in *rd1* whole retina at post-natal day (P)13**

**Supplemental Table 2 – Differentially expressed genes (DEGs) in *rd1* rod photoreceptors at post-natal day (P)13**

**Supplemental Table 3 – Differentially expressed genes (DEGs) in *rd1* cone photoreceptors at post-natal day (P)13**

**Supplemental Table 4 – Ca^2+^-related GO terms in rod photoreceptors**

**Supplemental Table 5 – Ca^2+^-related GO terms in cone photoreceptors**

**Supplemental Table 6 – Quantification of TUNEL positive, dying cells in outer nuclear layer (ONL).**

**Supplemental Table 7 – Quantification of cells positive for calpain activity/activation in outer nuclear layer (ONL).**

**Supplemental Table 8 – Quantification of cells positive for PARP activity, PAR accumulation, and sirtuin activity in outer nuclear layer (ONL).**
